# High-performance Mg–Zn alloy achieved by the ultrafine grain and nanoparticle design

**DOI:** 10.1016/j.bioactmat.2024.07.020

**Published:** 2024-07-27

**Authors:** Wenhui Wang, Xiyue Zhang, Anke Zhang, Han Yu, Xinbao Kang, Cheng Wang, Yang Song, Jiahua Ni, Mikhail L. Zheludkevich, Xiaonong Zhang

**Affiliations:** a State Key Laboratory of Metal Matrix Composites, School of Materials Science and Engineering, Shanghai Jiao Tong University, Shanghai, China; b College of Biological Science and Medical Engineering, Donghua University, Shanghai, China; c The Second Affiliated Hospital, Zhejiang University, Zhejiang, China; d Institute of Surface Science, Helmholtz-Zentrum Hereon, Geesthacht, 21502, Germany; e Suzhou Origin Medical Technology Co. Ltd., Jiangsu, China

**Keywords:** Biodegradable metal, Mg alloy, Ultrafine grains, Nanoparticles, ECAP

## Abstract

Improving the comprehensive performance of low alloyed Mg is a significant challenge for biomedical applications. This paper developed a high-performance Mg–Zn alloy with uniform ultrafine grains and nano-precipitates through a straightforward, high-temperature reciprocating equal channel angle extrusion (ECAP) process and researched the microstructure, mechanical property, degradation behaviour, and biocompatibility of the developed alloy. Results showed that the lean Mg–2Zn alloy successfully refined grain to about 1 μm and produced plenty of nano-particles with uniform distribution, providing high comprehensive mechanical properties (YS: 235 MPa, UTS: 267 MPa, EL: 15.6 %). Additionally, Zn-riched nano-particles in the matrix could decrease the Zn aggregation at the corrosion layer-matrix interface and form a dense oxide film, achieving a low degradation rate (0.13 mm/year *in vivo*). Finally, this work realizes the low alloy content, low cost, and good properties of one biodegradable Mg alloy, which will benefit the promotion of future clinical applications.

## Highlights

• A lean biodegradable Mg–Zn alloy was developed through an efficient reciprocating and high-temperature ECAP technology.The simple alloy and good comprehensive performance can benefit subsequent medical device registration.

## Graphical abstract

**Figure:**
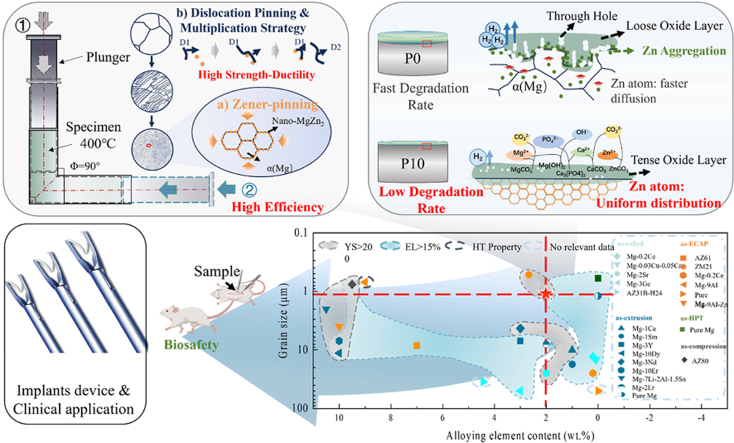

## 1 Introduction

Magnesium and its alloys have good biodegradability and biocompatibility, possessing great potential in absorbed implants in many medical fields [[1], [2], [3]]. Nonetheless, Mg's low formability, low mechanical properties (compared with traditional medical metals), and rapid degradation impede their industrialized manufacture and further clinical application [[4], [5], [6], [7]]. The lean Mg alloy with a simple chemical composition and low alloy content can balance mechanical properties, degradation rate and biological safety to a certain extent [8,9]. However, the high strength-ductility synergy of Mg alloys still depends on multiple types and the high content of alloying elements (especially RE elements) at the present stage [10]. Therefore, developing the theory and technology to improve lean Mg alloy's strength and ductility is crucial for biomedical applications.

Severe plastic deformation (SPD) is one of the most widely used methods to increase comprehensive mechanical performance through significant grain refinement [11,12]. However, due to Mg's hexagonal close-packed (HCP) structure, its singular independent slip system would result in poor ductility. Thus, it is prone to crack during SPD and challenging to accumulate high dislocation densities to refine grain availably. But at high temperatures and Zn addition, the deformability can be significantly promoted based on the activation of the pyramidal **<c+a>** dislocation slip in Mg alloys [13,14]. Our previous works found that the lean Mg–2Zn alloy can achieve an intense deformation under high temperatures rolling and induce efficient dynamic recrystallization (DRX) [15]. Partial nanocrystals can even be obtained under Zn segregated at grain boundaries through significant rolling deduction [16]. However, the elongation of the rolled Mg–2Zn alloy in previous work was still not expected due to the excessive grain boundary strengthening and firm texture after hot rolling, which may limit further use.

ECAP is a suitable technique to refine grain and cause texture softening to improve ductility [13]. The critical limitation of ECAP in commercial manufacture is the inefficiency in processing. In addition, the applications of ECAP need a degree of ductility for Mg alloys, which is usually researched in complex composition Mg alloys such as AZ31, AZ91, Mg-RE alloys and Zr-contented Mg alloys [[17], [18], [19]]. Thus, using ECAP to develop a lean biomedical Mg alloy is confronted with the technology's feasibility.

This research developed a specific reciprocating hot ECAP for the lean Mg–2Zn alloy to obtain uniform ultrafine grain, dispersive nano-particles and weak texture. The designed technology is based on the high-temperature-induced **<c+a>** slip in Mg–2Zn alloy under large plastic deformation. Consider nano-precipitates can act as both sources and barriers to dislocation motion under high-stress conditions, resulting in sustained strain hardening deformation and enhanced plasticity and strength [20]. The study designs nano-particles to be produced under dynamic segregation and precipitation during severe strain to hinder the coarsening of grains at high temperatures. Therefore, through the ECAP processes, grain refinement, nano-particles, and weakened texture can be achieved by accumulating significant strain through repetitive extrusion without altering the material's dimensions. The SEM (Scanning Electron Microscope), EBSD (Electron Backscatter Diffraction), TEM (Transmission Electron Microscope) and AR-TEM (Spherical Aberration Corrected Transmission Electron Microscope) were used to characterize the microstructure and morphology of Mg–2Zn alloy during the ECAP processes. The tensile tests, VPSC (Visco-plastic Self Consistent) simulations, 3D-XRM (X-ray Microscope) and electrochemical tests were used to evaluate mechanical properties and corrosion resistance. Furthermore, animal experiments were conducted to evaluate the *in vivo* degradation behaviour and biocompatibility. This paper aims to establish a scientific theory for creating highly fine-grained lean magnesium alloys with outstanding properties and explore definite technology for the application.

## 2 Experimental procedures

### 2.1 Materials & design

**Fig. 1:**
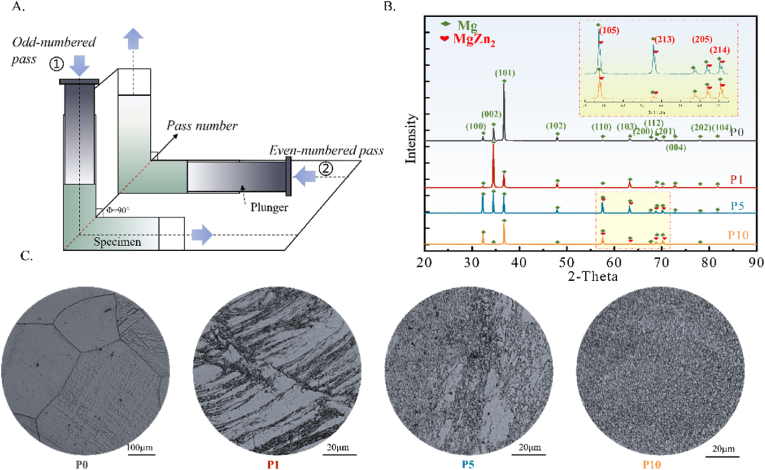
(A) ECAP process of Mg–2Zn alloy; (B) X-ray diffraction patterns; (C) metallographic morphology of ECAP prepared samples 10 $\bar{1}$ 0.

***ECAP procedure*** The solid-solution Mg–2Zn (wt.%) alloy (purity 99.98 %) with the dimensions of 15 × 15 × 50 mm was provided by (Suzhou Origin Medical Technology Co., Ltd., Suzhou, China). The solution treatment for the alloy ingot is 4 h at 340 °C. Multi-pass deformation was performed using the Equal Channel Angular Pressing (ECAP) process, where the specimen underwent reciprocating extrusion along the internal path of the die between different passes, as shown in Fig. 1A. The ECAP die-set includes internal and external mould (Fig. S1). The internal mould provides the deformation route for samples, and the external mould helps to keep the temperature. After finishing ECAP, the internal mould can be pulled out, separating Part 1 and Part 2 to move the sample without fracture. The effective deformation part is about 40 mm when the sample is initially 60 mm long (the initial size of the samples is 15 × 15 × 60 mm and the final size of the effective sample is about 15 × 15 × 40 mm). The samples were subjected to treatment at 400 °C for 1 h and then deformed in the ECAP die at a pressing rate of 10 mm/s for the first pass. After deformation, the punch was placed at the exit port, and the sample's reverse extrusion for the second pass was carried out at the same rate. The reciprocating extrusion path adopted in this paper under high-temperature conditions is similar to route A in a recent report [21]. However, the studied path does not need to remove the material after every pass, which has higher processing efficiency. The ECAP mould channel had an internal corner angle (*ϕ*) of 90° and an outer radius (*ψ*) of 0°. The strain per pass was determined to be 1.15 based on the Iwahashi theory [22].

(1a) $$\varepsilon =\frac{1}{\sqrt{3}}\left[2\;\cot\left(\frac{\phi }{2}+\frac{\psi }{2}\right)+\psi cosec\left(\frac{\phi }{2}+\frac{\psi }{2}\right)\right]$$

The Mg-2wt%.Zn alloy was subjected to 0, 1, 5, and 10 pressing passes and quenched, respectively. The samples were named P0, P1, P5, and P10. The transeverse direction (TD) plane of all samples is selected for microstructure characterization and corrosion resistance tests.

***XRD*** X-ray diffraction (XRD) analysis was performed using a D8 ADVANCE Da Vinci type XRD instrument (Bruker, Germany) with Cu Kα radiation (λ = 0.154056 nm). The scanning of the sample phase was conducted at 50 kV, 300 mA, 20°–90° scanning angle (2θ), and a scanning speed of 4°/min. The XRD data was processed and analyzed by using Jade 6 software.

***Metallographic structure*** Samples were polished and ground, followed by preliminary microstructure observation using a metallographic optical microscope.

### 2.2 Mechanical properties

***Hardness*** The samples were ground, and the microhardness of each sample was measured on a digital hardness tester. The load was 9.8 N, with a loading time of 10s. The number of parallel samples was n = 2, and 3 points were collected for each sample.

***Tensile test*** The miniaturized dog bone-shaped sample possessed an overall length of 32 mm for mechanical tests. This test sample's gauge length and width are 10 mm and 2 mm, respectively. The direction of tensile deformation is parallel to the extrusion direction. Tensile tests were conducted at RT on an Instron-8872 universal testing machine at a 0.15 mm/min rate. The final data of tensile tests were based on the average result of three samples.

***VPSC plastic simulation*** To understand the Influence of grain size on the plastic deformation process, the visco-plastic self-consistent (VPSC) model (version 7d) was employed to simulate the activation of slip systems in the grain interior during the plastic deformation stage of the P1 and P10 samples. The VPSC procedure is used to predict the local and overall viscoplastic response of a polycrystal as: $U_{i,j}=E_{ij}+W_{ij}$, in which $U_{i,j}$ represents velocity gradient, $E_{ij}$ is the symmetric strain-rate and $W_{ij}$ is the skew-symmetric rotation-rate. At the local level in a polycrystalline aggregate, the visco-plastic constitutive behavior is characterized by the non-linear rate sensitivity Eq (1a), (1b):

(1b) $$\varepsilon_{ij}\left(\bar{x}\right)=\sum_{s}m_{ij}^{s}\gamma^{s}\left(\bar{x}\right)=\gamma_{o}{\sum_{s}\left(\frac{m_{kl}^{s}\sigma_{kl}\left(\bar{x}\right)}{\tau_{o}^{s}}\right)}^{n}$$

in which $\tau^{s}$ and $m_{ij}^{s}=\frac{1}{2}\left(n_{i}^{s}b_{j}^{s}+n_{j}^{s}b_{i}^{s}\right)$ are the threshold stress and the symmetric Schmid tensor associated with slip (or twinning) systems. In the above expression, ${\bar{n}}^{s}$ is the normal vector and ${\bar{b}}^{s}$ is the Burgers vector of slip (or twinning) system, $\varepsilon_{ij}\left(\bar{x}\right)$ and $\sigma_{kl}\left(\bar{x}\right)$ are the deviatoric strain-rate and stress, and $\gamma^{s}\left(\bar{x}\right)$ is the local shear-rate on slip systems.

In the above function, the threshold stress describes the activation resistance experienced by the deformation modes. Its value usually increases with deformation and is defined by Voce hardening mode (2) [23]:

(2) $${\hat{\tau }}^{s}=\tau_{0}^{s}+\left(\tau_{1}^{s}+\theta_{1}^{s}\Gamma \right)\left(1-\exp\left(-\Gamma \left|\frac{\theta_{0}^{s}}{\tau_{1}^{s}}\right|\right)\right)$$

where $\Gamma =\Sigma \Delta \Upsilon^{s}$ represents the accumulated shear stress in the grains ($\Delta \Upsilon^{s}$ is the threshold stress), $\tau_{0}$ is the initial critical resolved shear stress (CRSS), $\theta_{0}$ is the initial hardening rate, $\theta_{1}$ is the saturation hardening rate, and $\left(\tau_{0}+\tau_{1}\right)$ represents the critical resolved shear stress obtained using the back-extrapolation method. These parameters were adjusted until the experimentally obtained stress-strain curves and pole figures matched with the simulated ones. The velocity gradient component during the tensile process is (3)

(3) $$\dot{L}=\left[\begin{array}{ccc} -0.5\nu & 0 & 0\\ 0 & -0.5\nu & 0\\ 0 & 0 & \nu \end{array}\right]$$

in which ν represents the strain rate along the z-axis. The initial 2500 grains' orientation (Euler angles and fractions) were determined from EBSD results at the middle of the standards tensile samples before tensile deformation.

### 2.3 Microstructural analysis

***EBSD*** P1, P5, and P10 samples were performed using an Extreme-resolution Analytical Field Emission Scanning Electron Microscope (Tescan Mira 3 XH, Tescan, Czechia) equipped with an Electron Backscatter Diffraction (EBSD) system. EBSD results were post-processed using Channel 5 software.

***TEM & AC-TEM*** The TEM (Talos F200X G2, Thermo Fisher Scientific, USA) and AC-TEM (JEM-ARM300F) were utilized to observe the microstructure and elemental distribution of samples P1 and P10. Sample preparation methods were performed following previous studies [15].

### 2.4 Corrosion measurement

***Electrochemical testing*** Electrochemical tests were conducted using a PARSTAT-2273 electrochemical system. The size of cuboid samples for the electrochemistry test is 15 × 15 × 2 mm. The electrode system consisted of a working electrode (sample, n = 3), an Ag/AgCl reference electrode as the reference electrode, and a platinum (Pt) as the counter electrode. The electrodes were immersed in Hank's solution with a pH of 7.4 (excluding Mg^2+^, Ca^2+^, and phenol red indicator), and the temperature was maintained at 37 ± 1 °C. The open circuit potential was first stabilized for 3600 s, followed by impedance spectroscopy measurements from 10^5^ Hz to 10^−1^ Hz with an AC amplitude of ±5 mV. Subsequently, a potential dynamic test was conducted by scanning from −0.5 V_OCP_ to 0.5 V_OCP_ at a scan rate of 0.1 mV/s. ZsimDemo software was used to fit the impedance spectra using relevant equivalent circuits.

***In vitro degradation testing*** To investigate the corrosion morphology and elemental composition of formed corrosion products distribution after immersing in Hank's solution for 24 h at 37 °C, the samples P0 and P10 were dried and subjected to focused ion beam (FIB) milling using an FEI Tecnai G2 F20. Lift-out processing was performed to extract thin sections from the sample surface, and the immersion sample cross-section was characterized using FEI Talos F200X Scanning Transmission Electron Microscopy (STEM).

***In vivo degradation testing*** Three 4-month-old male mice were provided by the Second Affiliated Hospital, Zhejiang University. P0, P1, P5, P10, and high-purity magnesium (HPM) samples (1 × 1 × 5 mm) were implanted into the abdominal cavity of mice. After 1 week, the samples were retrieved. The surface morphology of the specimens was reconstructed in three dimensions using X-ray microscopy (Xradia 520 Versa, Carl Zeiss, Germany) (Pixel sizes: 4.8346, detector distance: 8 mm, source distance: 20 mm, exposure time: 0.25 s, voltage: 80 kV). During the scanning process, a filtered secondary reference was applied to modify the beam for the reference and tomography scans to reduce diffuse artefacts in the metal implant images. The Dragonfly 4.1 software (Object Research Systems, USA) was used to reconstruct the three-dimensional images of the implants. The corroded samples were ultrasonically cleaned in a solution containing 180 g/L chromic acid and 10 g/L AgNO_3_ for 1 min to remove surface products. Then, the surface was cleaned with water and absolute alcohol and weighed. The corrosion rate was calculated using the following formula (4) [15],

(4) $$Corrosion\;rate\;\left(mm/year\right)=\frac{8.76\times 10^{4}\times W}{T\times A\times \rho }$$

where W represents the loss weight (g), T is the degradation time (day), A is the exposed surface area (cm^2^), and ρ is the material density (1.74 g/cm^3^). All animal experiments were conducted according to the Guidance Suggestions for the Care and Use of Laboratory Animals (issued by the Ministry of Science and Technology of the People's Republic of China) and approved by the Animal Care and Experiment Committee of the Second Affiliated Hospital, Zhejiang University.

### 2.5 Inflammatory response

***Histological analysis*** Tissues were fixed overnight in 10 % neutral buffered formalin (Sigma-Aldrich, HT501128-4L) and embedded in paraffin. 8 μm tissue sections were stained with Hematoxylin and eosin (Beyotime, C0105S) according to the manufacturer's instructions. For immunohistochemistry (IHC), rehydrated tissue sections were blocked with goat bovine serum overnight at 4 °C and stained with CD68, CD3, CD45, IL6 and TNFα. After washing, the sections were incubated with biotinylated anti-mouse IgG or anti-rabbit IgG (Vector Laboratories, CA, USA). The ABC method (Vector Laboratories) was used with 3,3′ diaminobenzidine (Dojindo Laboratories, Kumamoto, Japan) as a substrate for detection. The sections were observed using an AX-80 microscope (Olympus, Tokyo, Japan).

***Real-time quantitative PCR (RT-qPCR)*** Total RNA was isolated from tissue samples with TRIZOL Reagent and reverse transcribed by PrimeScript RT Master Mix kit (Takara, Japan) according to the manufacturer's protocol. mRNA expression was determined and quantified through 2−ΔΔCT. The primer sequences are listed in Supplementary Table 1.

### 2.6 Statistical analysis

The results were expressed as mean ± standard deviation (SD). Statistical significance was analyzed by unpaired T-test of prime software, which was used to compare the p values of different groups during animal testing, and the differences were considered statistically significant when *p < 0.05.

## 3 Results

### 3.1 Microstructures and mechanical properties

According to the XRD results (Fig. 1B), it can be seen that P0 and P1 samples mainly exist in a single phase of α(Mg), with a firm texture orientation along (101) and (002), respectively. As the ECAP pass number increases, the matrix texture strength in the Mg–2Zn alloy decreases, and a minor peak of the second phase MgZn_2_ is detected between 55° and 70° (as shown in the magnified image). Metallographic results (Fig. 1C) show that the Mg–2Zn alloy has a coarse microstructure after solid solution. After 1 ECAP pass, the P1 sample exhibits typical deformation characteristics, such as enlarged grains, elongation deformation, and abundant twins. As ECAP passes increases, the coarse deformation microstructure decreases, and the proportion of refined equiaxed grains increases. By 10 ECAP passes, the P10 sample consists entirely of refined and uniform equiaxed grains.

**Fig. 2:**
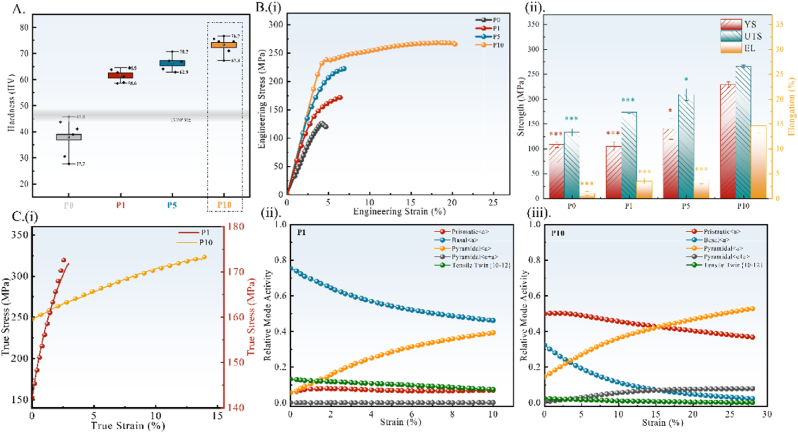
(A) Vickers hardness; (B) (i) Strain-stress curves; (ii) strength and elongation values obtained from the curve; (C) the VPSC simulation results: (i) the experimental and simulated true strain-stress curves of P1 and P10 samples; (ii-iii) the relative activation of different deformation modes of P1 and P10 samples. Data were expressed as mean ± SD. n = 3 in panels *: p < 0.05, **: p < 0.01, ***: p < 0.001.

The surface Vickers hardness values (Fig. 2A) of the P1, P5, and P10 samples are 61.63 ± 2.50 HV, 66.34 ± 3.03 HV, and 73.3 ± 3.51 HV, respectively, which were all significantly higher than the solid solution Mg–2Zn (37.97 ± 7.31 HV) and ECAP-HPM (46.23 ± 2.21 HV). The stress-strain curve during the tensile process is shown in Fig. 2B(i). With an increase in processing passes, the Mg–2Zn alloy exhibits higher yield stress (YS), ultimate tensile strength (UTS), and elongation (EL). The data obtained from the stress-strain curve are shown in Fig. 2B(ii), where P10 shows a significant improvement in YS (235 MPa), UTS (267 MPa), and EL (15.6 %) compared to P0 (103 MPa, 127 MPa, 1.3 %), P1 (116 MPa, 172 MPa, 3 %), and P5 (164 MPa, 223 MPa, 2.9 %).

To further investigate the plastic deformation mechanism during the tensile process, the initiation of slip systems in the plastic deformation stages of P1 and P10 was simulated and analyzed using the VPSC model. The simulated results are shown in Fig. 2C (i), and the plastic deformation curve obtained by VPSC simulation shows a good fit with the experimental data, indicating that the simulation result is close to the actual plastic deformation process. Fig. 2C (ii) and (iii) display the activation of slip systems for P1 and P10, respectively. During the initial stage of plastic deformation, P1 mainly undergoes basal <**a**> slip (proportion: 75.4 %) and gradually reduces as the strain increases, while pyramidal <**a**> slip proportion increases. With an increase in the processing passes, the deformation mode of each slip system in P10 changes significantly at the initial stage of plastic deformation. At this point, compared to P1, the proportion of basal <**a**> slip (32.3 %) is half decreased, and prismatic <**a**> slip (49.9 %) becomes the primary mode of plastic deformation. As the strain increases, pyramidal <**a**> slip gradually becomes the dominant way (52.8 %), while prismatic <**a**> slip decreases to 36.8 %. It is worth noting that during the P10 deformation, pyramidal **<c+a>** slip is activated, and final accounting for 7.9 %. It contributes to enhancing the plastic deformation capability of the Mg–2Zn alloy under RT conditions. The increase in non-basal slip is mainly attributed to changes in the CRSS between basal and non-basal slip systems $\Delta \tau_{0}$ [24]. According to the VPSC results, as ECAP passes increase, $\Delta \tau_{0}$ between basal <**a**> slip and prismatic <**a**> slip decreases from 102 MPa to 90 MPa, while $\Delta \tau_{0}$ between basal <**a**> slip and pyramidal **<c+a>** slip decreases from 120 MPa to 100 MPa. The VPSC simulation results confirmed that the ECAP reciprocating extrusion process could promote the activation of non-basal slip systems and improve the plastic deformation capability of the Mg–2Zn alloy.

**Fig. 3:**
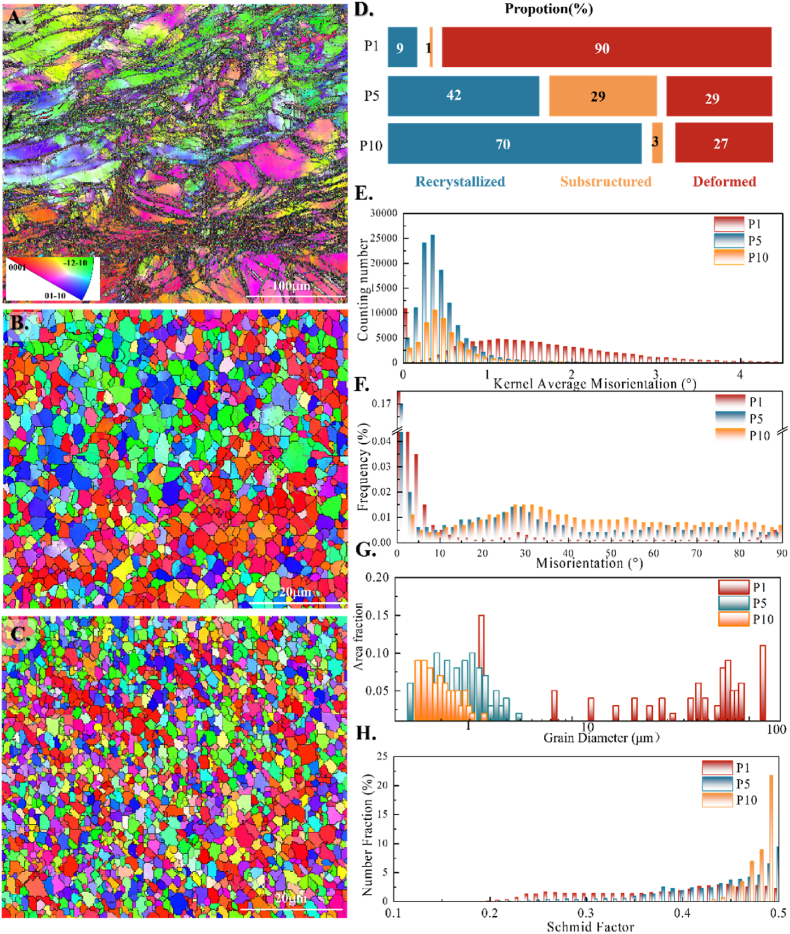
EBSD inverse pole figure (IPF) maps of (A) P1, (B) P5, (C) P10 (the colour map representing the grain orientation of each sample is indicated in the inserted crystallographic axes); (D) the proportion of deformed, sub-structured and recrystallized grains; (E) the distribution of local misorientation; (F) the distribution of grain boundary misorientation; (G) the distribution of grain size of P1, P5 and P10 samples; (H) the Schmid factor distribution of **<c+a>** slip systems.

EBSD results of P1, P5 and P10 are shown in Fig. 3. From the inverse pole figure (IPF), it can be observed that after 1 pass of deformation (Fig. 3 A), a large amount of deformed twinned grains and deformed grains appeared, with intertwined twinned boundaries and curved grain boundaries. Small dynamically recrystallized grains were present at the boundaries, forming a “necklace” structure, and the grain size exhibited a bimodal distribution. At 5 passes (Fig. 3 B), the dynamically recrystallized grains at the grain boundaries grow more extensive, and the coarse deformed grains further fragment, indicating a more significant grain refinement. With increasing deformation, recrystallized grains nucleate homogeneously and grow continuously at 10 passes (Fig. 3C), resulting in uniform equiaxed grain refinement. The proportions of deformed grains, substructured grains, and recrystallized grains were statistically analyzed, and the results are shown in Fig. 3D. After 1 pass, deformed grains account for 90 % of the material. At 5 passes, the proportions of substructured grains (29 %) and recrystallized grains (42 %) increased, while the deformed grains (29 %) decreased. With further accumulated deformation, at 10 passes, the proportion of substructured grains (3 %) decreases significantly, while the dominance of recrystallized grains (70 %) within the material becomes evident.

Kernel average misorientation (KAM) results are shown in Fig. 3E. A higher KAM value indicates severe plastic deformation. For recrystallized grains, a lower KAM value corresponds to a lower internal defect density [25]. The KAM value distribution of P1 is more dispersed, indicating an inhomogeneous deformation. As ECAP passes increase, the KAM value is distributed between 0° and 1°, and the counting rate decreases, indicating that the defect density decreases and the DRX ratio in the region increases. The result is consistent with the statistical results of the proportion of recrystallized grains.

According to the distribution of grain boundary misorientation (Fig. 3F), the proportion of low-angle boundaries (LABs, 2° < misorientation <15°) in P1 is much higher than that of high-angle boundaries (HABs, misorientation >15°). As the process increases, the LABs within the alloy gradually transform into HABs. The grain size distribution is shown in Fig. 3G. In P1, the grain size exhibits a typical bimodal distribution, with a maximum grain size of up to 68.98 μm. In contrast, the P10 grains are significantly refined, with an average grain size of about 1 μm. Fig. 3H displays the Schmid factors of the **<c+a>** slip systems, with P10 having the highest Schmid factor, followed by P5 and P1. As the number of processing passes increases, the material becomes more susceptible to **<c+a>** slip, indicating higher plasticity, consistent with the results of tensile experiments and VPSC simulations.

Based on the EBSD results, it can be inferred that during the initial deformation stage (P1), the material undergoes uneven large plastic deformation. DRX occurs at the shear bands and twin boundaries, forming a “necklace” structure. With the increase of deformation passes (P5), DRX occurs continuously inside the material. The texture strength is significantly weakened. The dislocation is rearranged in the grains and forms a subcrystalline structure, which leads to the further division of the coarse, deformed grains. At 10 passes (P10), the subgrains absorb mobile dislocations, and the subgrain boundaries gradually evolve into high-angle grain boundaries, forming uniform and small recrystallized grains throughout the material.

**Fig. 4:**
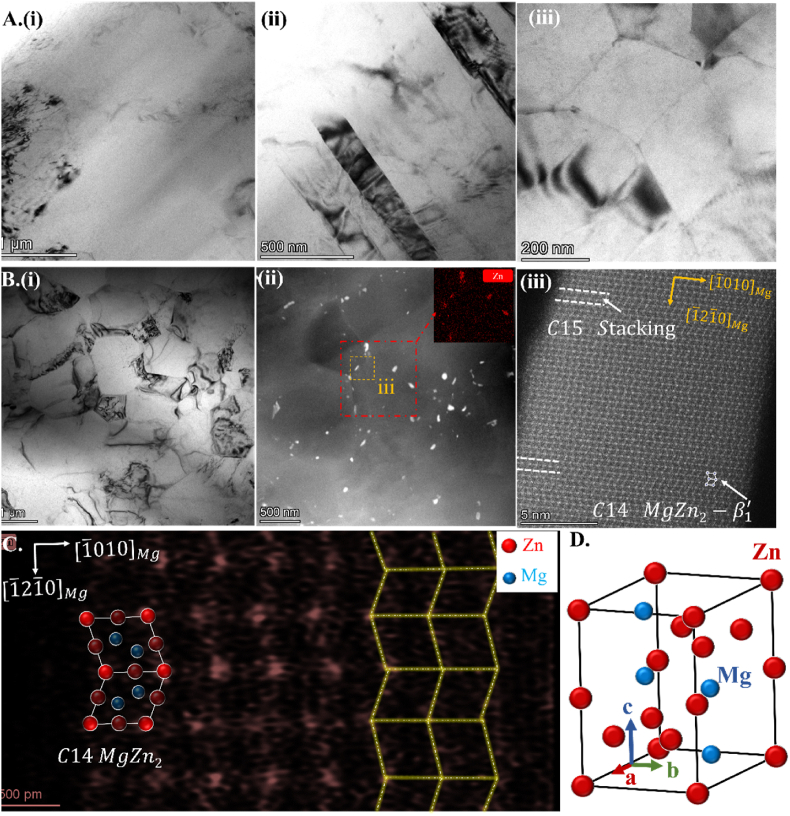
(A, i-iii) Bright-field TEM images of P1; (B) STEM images of P10 (i) bright-field images, (ii) HAADF-STEM, (iii) HRTEM images of secondary phase; (C) EDX mapping of HRTEM image of secondary phase and its unit cell structure in (D). the electron beam is parallel to [0001]_Mg_.

TEM and AC-TEM results further reveal the microstructure of P1 and P10. The microstructure of P1 consists of coarse deformed grains (Fig. 4 A(i)), deformed twins (Fig. 4 A(ii)), and nanoscale dynamically recrystallized grains (Fig. 4 A(iii)). After 10 passes of ECAP deformation, the P10 grains undergo equiaxed refinement (Fig. 4 B(i)) as observed in HAADF-STEM images (Fig. 4 B(ii)). Additionally, nanoscale Zn precipitation phases are present. According to the statistics in the STEM image, these particles' average diameter and volume fraction are about 48.67 nm and 2.36 %, respectively. Most precipitation phases are located at grain boundaries and exhibit a lath-shaped morphology. A few rod-shaped precipitation phases exist within the grains, possibly related to the lack of interfaces [26]. HRTEM analysis was performed on the lath-shaped precipitation phases within the yellow dashed-line box at the grain boundaries to determine the primary composition of these precipitation phases. The results show (Fig. 4 B(iii)) that the Zn precipitation phases at the interfaces exhibit typical Laves $Mg{Zn}_{2}-\beta_{1}^{’}$ structure features and are coherent with the Mg matrix [26]. The atomic scale resolution investigation of lath-shaped β₁' enables clear identification of the C14 (hexagonal phase) and C15 (cubic phase) Laves phase variants, which are built up by (0001) plane stacking parallel to ${\left[\bar{1}2\bar{1}0\right]}_{Mg}$ direction. EDX mapping under HRTEM reveals that the Zn atoms in the C14 $Mg{Zn}_{2}-\beta_{1}^{’}$ structure (Fig. 4C) grow in a wave-shaped manner along the ${\left[10\bar{1}0\right]}_{Mg}$ direction (yellow line), as depicted in the atomic ball-stick model shown in Fig. 4D.

### 3.2 Degradation testing

**Fig. 5:**
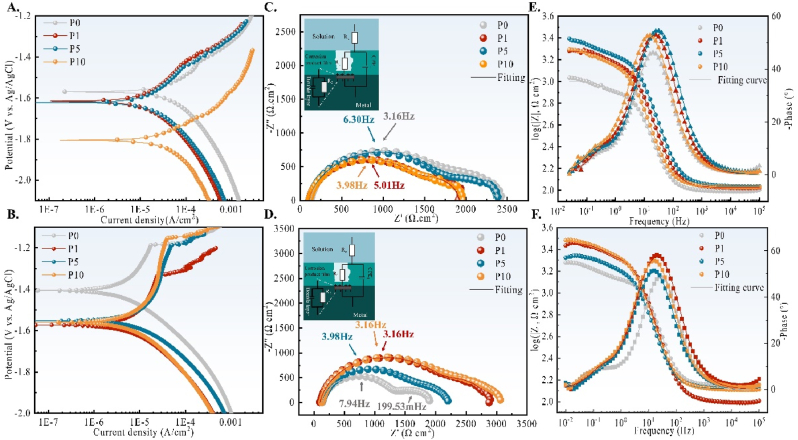
Potential dynamics polarisation curves of (A) 1 h and (B) 24 h immersion in Hank's at 37 °C and pH = 7.4; (C) 1 h and (D) 24 h Nyquist spectral curves and the schematic diagram of fitted equivalent circuits, where *R*_*sol*_, *R*_*1*_, *CPE*_*1*_, *R*_*2*_ and *CPE*_*2*_ denote solution resistance, film layer resistance, film layer capacitance, charge transfer resistance and double layer capacitance, respectively; (E) 1 h and (F)24 h Bode plots.

Sample degradation performance was evaluated using 1 h and 24 h potentiodynamic polarisation (PD) curves and electrochemical impedance spectroscopy (EIS). Fig. 5(A) and (B) illustrate the polarisation curves of the samples after 1 h and 24 h immersion in Hank's solution, respectively. The hydrogen evolution reaction controls the cathodic branch with a small contribution to the dissolved oxygen reduction [27]. At the same time, the anodic polarisation curve reflects the dissolution reaction of Mg. The corrosion current density (I_corr_) obtained from the polarisation curves is shown in Table 1. At 1 h, the cathode corrosion currents (calculated as Ref [28]) for P0, P1, P5, and P10 are 5.20 $\times 10^{-5}$ mA/cm^2^, 1.79 $\times 10^{-5}$ mA/cm^2^, 2.39 $\times 10^{-5}$ mA/cm^2^ and 4.56 $\times 10^{-5}$ mA/cm^2,^ respectively, indicating higher dissolution rates for P10 and P0. After 24 h immersion in Hank's solution, the anodic polarisation curves demonstrate a distinct breakdown potential, indicating the formation of a well-defined layer at the sample surface. The cathode corrosion currents for each sample are 6.31 $\times 10^{-6}$ mA/cm^2^, 9.99 $\times 10^{-6}$ mA/cm^2^, 1.36 $\times 10^{-5}$ mA/cm^2^ and 2.70 $\times 10^{-6}$ mA/cm^2^, with corrosion potentials of −1.41V, −1.57V, −1.55V, and −1.56V, respectively. The breakdown potentials and the differences between them and E_corr_ (ΔE) at 24 h are shown in Table 2. With increased ECAP passes, ΔE values increase, indicating denser corrosion layers and better substrate protection.

**Table 1:** Electrochemical parameters obtained from PD diagrams.

	P0	P1	P5	P10
***I***_**corr**_**-cathode (mA/cm**^**2**^**)**	5.20 E−5	1.79 E−5	2.39 E−5	4.56 E−5
***E***_**corr**_**(V vs. Ag/AgCl)**	−1.57	−1.62	−1.62	−1.81
***I***_**corr**_**-cathode (mA/cm**^**2**^**)**	6.31 E−6	9.99 E−6	1.36 E−5	2.70 E−6
***E***_**corr**_**(V vs. Ag/AgCl)**	−1.41	−1.57	−1.55	−1.56

**Table 2:** The breakdown potential of the barrier layer after 24 h

	P0	P1	P5	P10
*Breaking potential (V)*	−1.19	−1.33	−1.19	−1.15
*ΔE*（V）	0.22	0.24	0.36	0.41

Fig. 5(C) and (D) show the EIS spectra of the samples after 1 h and 24 h immersion in Hank's solution at 37 °C, respectively. A larger curve arc indicates a higher impedance value and better corrosion passivity of the sample. AC impedance data were simulated using an equivalent circuit model to further quantify the differences in degradation performance. The deviation values of $\chi^{2}$ were all smaller than 10^−3^, as shown in Tables S1 and S2. The equivalent circuit diagram is illustrated in the upper left of Nyquist figures, where *R*_*sol*_ represents the solution resistance, *R1* and *CPE1* represent the resistance and capacitance of the corrosion layer, respectively. *R2* and *CPE2* represent the double-layer interface's charge transfer resistance and capacitance. *n* is the CPE coefficient shows how much CPE deviates from an idea capacitor. The electrode impedance *Z*_*w*_ and polarisation resistance *R*_*p*_ can be determined as follows [29]:

$$R_{p}=lim_{\omega \to 0}\left(Z_{w}\right)=lim_{\omega \to 0}\left(\frac{1}{\frac{1}{R_{1}}+\frac{1}{\frac{1}{R_{2}}+{CPE}_{2}{\left(jw\right)}^{n}}+{CPE}_{1}{\left(jw\right)}^{n}}\right)=R_{1}+R_{2}$$

Where ω is the angular frequency. After 1 h immersion in Hank's solution, the order of *R*_*p*_ values is as follows: P0 (2257.8 Ωcm^2^) > P5 (2229.2 Ωcm^2^) > P10 (1839.5 Ωcm^2^) > P1 (1806.4 Ωcm^2^). At 24 h, the order of Rp values is P10 (2907.6 Ωcm^2^) > P1 (2752 Ωcm^2^) > P5 (2031.4 Ωcm^2^) > P0 (1762.9 Ωcm^2^). *R1* and *R2* values follow the same order. The results indicate that, during early immersion (1 h), P10 exhibits a higher degradation rate. With prolonged immersion time, the protective oxide layer on the surface of the P10 sample improves, significantly reducing the substrate's degradation kinetics. According to Bode curves (Fig. E–F), all samples' impedance values |Z| rise from 10^5^ to 10^−2^ Hz. What's more, two wave crests in Phase vs. Frequency figures can be found, which verifies the existence of two capacitance loops in fitted equivalent circuits.

**Fig. 6:**
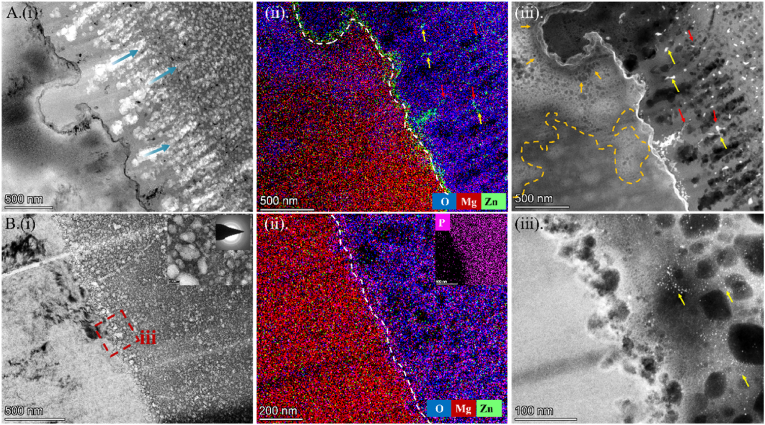
*In vitro* degradation results. Cross-sectional STEM images of the *in vitro* degradation products after 1-week implantation of (A) P0 and (B) P10 samples: (i) bright-field images, (ii) EDX mappings, (iii) HAADF-STEM images.

To further investigate the microstructure and elemental composition of the degradation products *in vitro*, the cross-sections of P0 and P10 samples soaked in 37 °C Hank's solution for 24 h were characterized by STEM. From the bright-field image (Fig. 6A (i)), it can be observed that the degradation of the P0 interface is uneven, with numerous interconnected pores along the growth direction of the oxide layer (blue arrows). The elemental distribution map (Fig. 6A (ii)) shows that Zn is enriched along the interface and forms clustered particles (yellow arrows) within the oxide layer, which are spatially correlated with the pores (red arrows). HAADF-STEM imaging (Fig. 6A (iii)) reveals that the average diameter of the pores within the corrosion product layer is 221.26 nm. In comparison, the average diameter of the Zn precipitation particles is 31.85 nm.

Furthermore, the matrix at the corroded pits on the interface appears more porous, with many pores along the interface (orange arrows), indicating a tendency for preferential degradation. It can be inferred that the Mg matrix will undergo significant degradation along the dashed orange line with prolonged immersion time. Compared to P0, the corroded interface of the P10 sample is smoother (Fig. 6B (i)), with the matrix structure well preserved and the pores within the barrier layer independent and denser, consistent with the electrochemical results. At this stage, Zn is dispersed within the barrier layer and the matrix (Fig. 6B (ii)). The average diameter of the pores within the corrosion product layer is 32.02 nm. In comparison, the average diameter of the Zn particles dispersed within the oxide film is 2.48 nm (Fig. 6B (iii)), varying by order of magnitude to the P0 sample. The cross-section STEM results of the immersed samples demonstrate that P10 has a denser corrosion product layer and provides stronger protection to the matrix than P0.

**Fig. 7:**
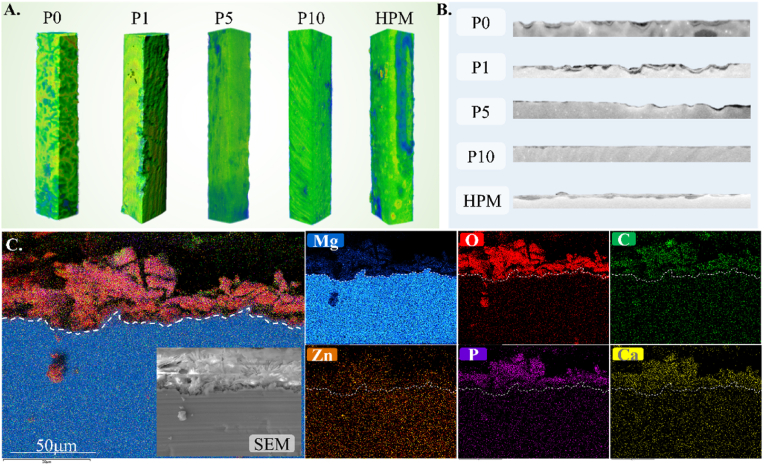
*In vivo* degradation results. (A) 3D reconstruction of *in vivo* degradation samples; (B) 2D cross-section images of *in vivo* degradation samples; (C)cross-sectional SEM and EDX images of the *in vivo* degradation products of P10 sample with 1-week implantation in SD rats.

Fig. 7A showed the surface morphology of P0, P1, P5, P10, and HPM samples after 1-week implantation in SD mice. Fig. 7B displayed the cross-sectional morphology. Compared to other samples, the surfaces of P10 and HPM are smooth, and their degradation is uniform. The weight loss results indicate the degradation rates within the samples as follows: P1 (0.20 mm/year) > P5 (0.19 mm/year) > P0 (0.15 mm/year) > P10 (0.13 mm/year) > HPM (0.10 mm/year). Fig. 7C exhibits the cross-sectional SEM and EDX of P10 after 1 week of degradation *in vivo*. According to SEM, the thickness of the degradation product layer on the surface of P10 is approximately 45 μm. The EDX spectrum reveals the presence of Zn, Mg, C, O, Ca, and P elements in the oxide layer, suggesting that the degradation product may consist of phosphates and carbonates of Zn, Ca, and Mg.

### 3.3 Inflammatory response/biosafety testing

**Fig. 8:**
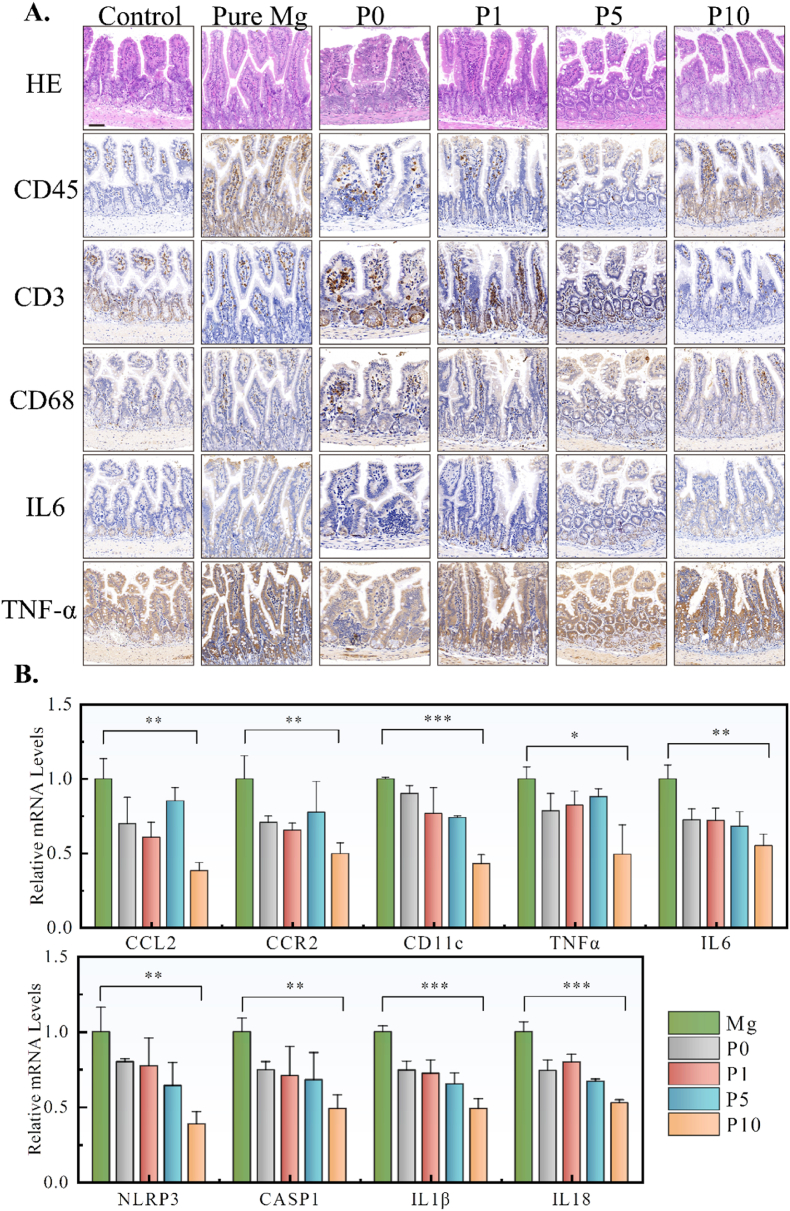
(A) Analyses of soft tissue slices of H&E staining and immunohistochemical staining of inflammatory factors (CD45, CD3, CD68, IL6, TNF-α) after 1-week implantation. (B) mRNA expression of CCL2, CCR2, CD11c, TNF-α, IL6, NLRP3, CASP1, IL1β, IL18. Data were expressed as mean ± SD. n = 3 in panels *: p < 0.05, **: p < 0.01, ***: p < 0.001.

According to the immunohistochemical staining (Fig. 8A), the infiltration of immune cells (CD3^+^ cells and CD68^+^ macrophages) and pro-inflammatory substances release (IL-6 and TNF-α) were alleviated with the implantation of P5 and P10 alloys. Consistent with the above results, P10 alloys also significantly inhibited the expression of proinflammatory (CCL2, CCR2, CD11c, TNFα, and IL6) and inflammasome-associated genes (NLRP3, CASP1, IL1β, and IL18) in intestinal tissues after implantation (Fig. 8B).

## 4 Discussion

This paper developed a convenient reciprocated ECAP technique to process the lean alloy of Mg–2Zn under high temperatures, obtaining a comprehensive increase in microstructures, mechanical properties, degradation behaviour and biocompatibility.

### 4.1 Technological parameter

Under normal circumstances, ECAP-treated Mg alloys have a weakened texture, reducing strength and improving ductility. Reducing the texture and refining the grain is necessary to enhance strength and ductility during the ECAP processes. The fundamental mechanism for forming Mg alloy texture during the ECAP process is the rotation of the dominant slip plane to be parallel to the theoretically activated shear plane during the ECAP process [[30], [31], [32]]. For N ≥ 2, different shear planes and slip systems are activated for different processing routes, resulting in closely related texture categories depending on the processing path [33]. The traditional ECAP processing routes are classified as *A*, *B*_*A*_, *B*_*C*_, and *C*, characterized by the angle and direction of sample rotation between consecutive passes [21]. The reciprocating extrusion path (*D*) adopted in this paper under high-temperature conditions is similar to A. Therefore, compared to the traditional routes, the *D* path does not need to remove the material, which has higher processing efficiency.

**Fig. 9:**
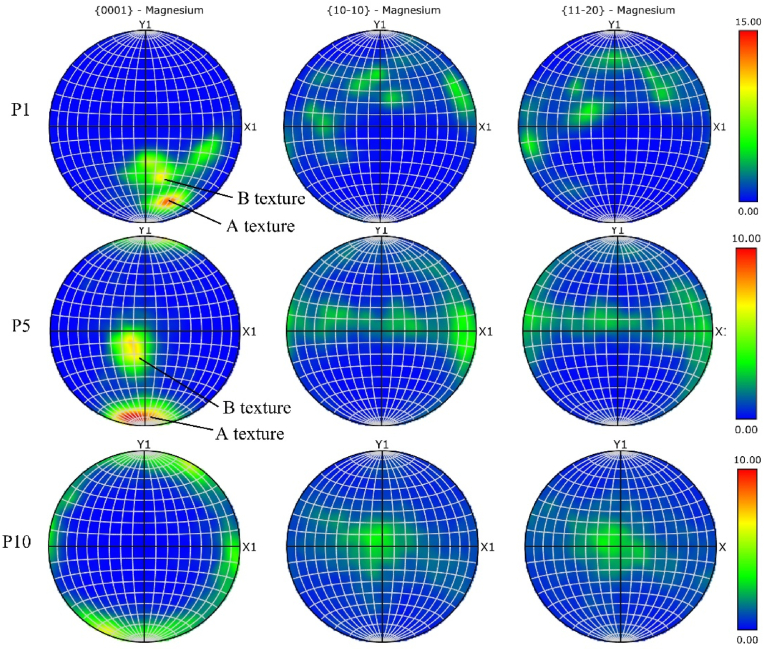
EBSD results of the pole figures of P1, P5, and P10 samples.

According to EBSD results, during the initial deformation stage (P1), the crystals undergo non-uniform large plastic deformation under high shear strain, forming a deformation texture within the material (Fig. 9). The texture comprises two components, T_A_ and T_B_. Component T_A_ may be related to the activation of a combination of twinning in compression and secondary pyramidal slip system $\left\{11\bar{2}2\right\}$. Component T_B_ may originate from the (0001) basal plane rotation [34]. As the pass number increases (P5), the texture strength decreases, with the component T_A_ strength decreasing from 12.9 to 11.0 and the component T_B_ strength decreasing from 9.9 to 7.1. There is a change in the texture type that deviates approximately 13° from the normal direction. The change is linked to the recrystallization and reconstitution of texture during the multi-pass (N > 2) extrusion process. As a result, the Influence of deformation texture is significantly reduced. At 10 passes, the T_B_ component disappears, and the T_A_ component strength decreases to 6.3. The tensile test results show that the P10 sample exhibits outstanding mechanical properties (YS 235 MPa, UTS 267 MPa, and EL 15.6 %). VPSC simulation results further reveal the activation of non-basal slip systems during the tensile process in P10 under room temperature, greatly enhancing the material's plasticity. Krajnak et al. also revealed that Mg alloys prepared through the A path tend to activate non-basal slip systems during tensile deformation. The interaction between non-basal and basal dislocations also leads to additional hardening [33].

Interestingly, unlike the strengthened T_A_ with the ECAP processes in the traditional A path, the T_A_ in this work decreased strength and deviated from the normal direction. The above changes should be attributed to the following reasons: i) The twinned grains (mainly tension twin) rotate along the basal planes to form angles of 86° with the axial direction, altering some of the orientations within the grains; ii) At high temperature, the activated **<c+a>** pyramidal slip system can provide more than 5 slip systems according to Taylor model, which can accommodate arbitrary deformation within the polycrystalline material. The increase in slip systems and the non-parallel activation of shear planes between different deformation passes make it more difficult for the dominating slip planes to rotate and form texture, thereby weakening the material's anisotropy; iii) DRX occurs during the ECAP process, and small recrystallized grains can rotate under the action of applied stress, which significantly weakens the texture [35]. The extrusion process (path + torsion angle + temperature) used in this paper can weaken the texture compared to the traditional process, eliminating material anisotropy. Therefore, the Mg–2Zn alloy can exhibit excellent comprehensive mechanical properties despite low alloying.

### 4.2 Grain refinement mechanism

Grain boundaries are a significant factor in improving the comprehensive mechanical properties of Mg alloy. As DRX induced by severe strain at high temperatures and nanoscale second-phase particles impede the growth of recrystallized grains, the Mg–2Zn grains undergo significant equiaxed refinement. According to the distribution of grain size results (Fig. 3G), the solid solution Mg–2Zn alloy has an average grain size of hundreds μm and is refined to about 1 μm with an ultrafine structure.

According to the *Arrhenius* relationship, the flow stress σ’(∝σ), strain rate $\dot{\varepsilon }$ and deformation temperature *T* in the metal materials plastic deformation process has the following manifestation [36]:

(6) $$\dot{\varepsilon }=C{\left[\sinh\;\left(\alpha \sigma {‘}^{\prime }\right)\right]}^{n}\;\exp\;\left(-\frac{Q}{RT}\right)$$

where C, α, n are material constants, $R=8.314\left(J\cdot {mol}^{-1}\cdot K^{-1}\right)$ is the gas constant, T is the absolute temperature, and Q is the hot deformation activation energy.

Based on the Zener-Hollomon parameter Z [37]:

(7) $$Z=\dot{\varepsilon }\exp\;\left(\frac{Q}{RT}\right)$$

where *Z* is a normalized parameter, which can well reflect the mutual compensation relationship between strain rate and temperature during deformation. Substituting Eq (7) into Eq (6):

(8) $$Z=\dot{\varepsilon }\exp\left(\frac{Q}{RT}\right)=C{\left[\sinh\;\left(\alpha \sigma^{\prime }\right)\right]}^{n}$$

Hence, the *Q* can be represented as:

(9) $$Q=Rn{\left[\frac{\partial \ln\left[\sinh\;\left(\alpha \sigma^{\prime }\right)\right]}{\partial \left(\frac{1}{T}\right)}\right]}_{\dot{ℇ}}$$

According to Eq (9), it can be observed that at the same $\dot{ℇ}$. A higher deformation *T* results in a larger *Q*, making the material more prone to DRX and leading to practical grain refinement [38,39]. What's more, the mechanical properties and grain sizes demonstrate a good agreement with the Hall-Petch relationship (5):

(5) $$\sigma =\sigma_{0}^{*}+kd^{-\frac{1}{2}}$$

where σ represents the yield stress, d represents the grain size, $\sigma_{0}^{*}$ and k are material constants. It illustrates that the alloy strength increases with the decrease in grain size, which is coherent with this paper's results.

### 4.3 Nanoscale secondary phase particles

According to the TEM results (Fig. 4) of the P10 sample, it observed the nano Laves phase ($Mg{Zn}_{2}-\beta_{1}^{’}$) near grain boundaries, which is in coherency with the Mg matrix. XRD spectra also exhibit small peaks of MgZn_2_ in the P5 and P10 samples. During the ECAP process, the strain-induced dislocations, vacancies and non-equilibrium grain boundaries can serve as nucleation sites for precipitates, which allows alloying elements to segregate to vacancies and reduce the effective activation enthalpy for diffusion and finally facilitates phase transformations within the matrix, promoting the formation of dynamic precipitation [40,41]. Yan et al. have confirmed that MgZn_2_ is a stable phase present in Mg–Zn alloy at temperatures ranging from 315 °C to RT. Moreover, MgZn_2_ precipitates in MgZn alloys typically exhibit rod/lath morphology and possess a certain crystallographic orientation relationship with the α(Mg) matrix, consistent with this study [40,42]. It is worth noting that the Laves $Mg{Zn}_{2}-\beta_{1}^{’}$' second phase, as a strengthening phase in Mg–Zn alloy, can not only contribute to precipitation hardening but also exhibit excellent plasticity [43]. It is speculated that the size of the second phase may also play a role besides grain refinement. The nano-sized particles can act as both dislocation sources and obstacles to dislocation motion under high stress, resulting in sustained strain hardening mechanisms and thus enhancing plasticity and strength [20,44].

### 4.4 Degradation and biosafety

*In vitro/in vivo* degradation studies have shown that P10 has a lower degradation rate under long-term immersion conditions. Generally, the degradation rate is related to grain size, initial potential, second phase size, and volume fraction [45]. The relationship between the degradation rate (*CR*) and grain size (*d*) of Mg alloys is similar to the Hall-Petch relationship [46]:

(5) $$CR=C+kd^{-\frac{1}{2}}$$

where k and C are constants. As the grain size decreases, the degradation rate slows down. The results confirm that P0 has a faster degradation rate due to coarse grain structure. It reflects numerous hydrogen pores within the corrosion products layer, which makes a looser film and causes non-uniform degradation at the matrix-corrosion layer interface. During the rapid degradation, the Zn element can accumulate at the interface, further promoting galvanic corrosion and creating a vicious cycle. In contrast, P10 samples with refined grains form a dense film on the surface, resulting in a reduced corrosion rate and uniform distribution of Zn within the matrix and layer. This behaviour significantly reduces the tendency for degradation and slows down the degradation rate of the matrix [47].

In clinical applications, it has been observed that there may be wear between implants and bone tissues during the implantation and service of metal implant devices. The worn particles can enter surrounding tissues and induce the aggregation of macrophages and secretion of chemotactic factors and pro-inflammatory cytokines, which result in an inflammatory phenotype and ultimately affect implant success rates [[48], [49], [50]]. According to the *in vivo* implantation experiment, P10 material exhibited high comprehensive mechanical properties, low degradation rate, and no inflammatory reactions during implantation. The inflammatory biomarkers were significantly lower than the control group, indicating good biocompatibility.

**Fig. 10:**
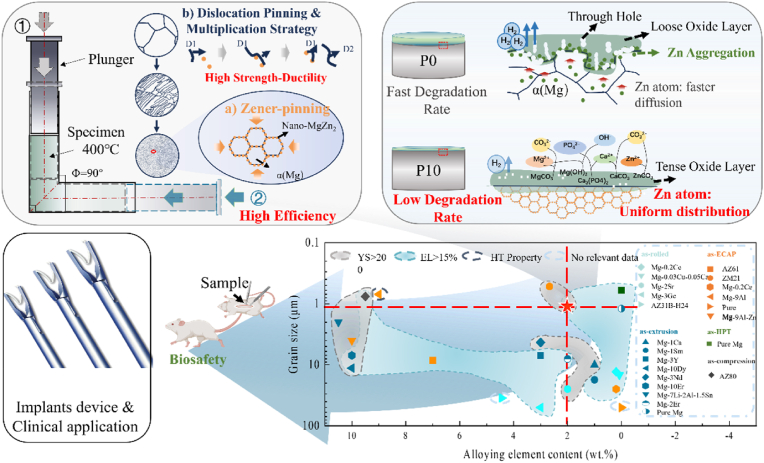
(A) The schematic diagram of ECAP technology and the degradation behaviour of prepared samples; (B) the comparison of grain size, alloy element content, plasticity and strength of P10 with typical recent studies^11-13,43-56^.

In summary, as shown in Fig. 10A, after the reciprocating extrusion of the ECAP process, the lean alloy of ultrafine Mg–2Zn with nano-precipitates was successfully developed. These nanoparticles can simultaneously enhance strength-ductility by providing Zener pinning to restrain the grain growth under HT and coordinate the obstruction and proliferation of dislocations. Compared to the rapid and uneven degradation of the alloy with solid solution treatment, which formed a loose and porous corrosion layer, the ultrafine grain structure exhibits a lower degradation rate. Additionally, Zn atoms, as components of MgZn_2_, are fixed within the matrix, preventing a preferential aggregation at the corrosion layer-matrix interface. As a result, the matrix degraded uniformly, leading to the formation of a dense oxide film. Fig. 10B compares alloying element contents and grain size of various Mg and its alloys fabricated by plastic deformation methods in typical recent studies [[17], [18], [19],[51], [52], [53], [54], [55], [56], [57], [58], [59], [60], [61], [62], [63], [64]]. Upon overall evaluation, the Mg–2Zn alloy possesses minimal alloy elements to achieve high biosafety, strength-ductility, low degradation, and excellent biosafety requirements, contributing to subsequent product registration and application.

## 5 Conclusions

This work successfully prepared the low alloyed Mg–2Zn with uniform ultrafine grains and nanoparticles using novel and efficient high-temperature reciprocating ECAP technology. The studied Mg–2Zn alloy exhibits good mechanical properties (YS: 235 MPa, UTS: 267 Mpa, EL: 15.6 %) due to the construction of the high density of grain boundary with nano-particles and good biocompatibility since appropriate degradation behaviour (0.13 mm/year *in vivo*), providing promising potential for subsequent device development and clinical applications.

## Ethics approval and consent to participate

All animal experiments were conducted according to the Guidance Suggestions for the Care and Use of Laboratory Animals (issued by the Ministry of Science and Technology of the People's Republic of China) and approved by the Animal Care and Experiment Committee of the Second Affiliated Hospital, Zhejiang University. Written informed consent was obtained from individual or guardian participants.

## CRediT authorship contribution statement

**Wenhui Wang:** Writing – review & editing, Writing – original draft, Investigation, Conceptualization. **Xiyue Zhang:** Writing – original draft, Software, Methodology, Investigation, Data curation. **Anke Zhang:** Methodology, Investigation. **Han Yu:** Methodology, Investigation. **Xinbao Kang:** Investigation. **Cheng Wang:** Writing – review & editing, Writing – original draft, Methodology, Investigation. **Yang Song:** Writing – review & editing. **Jiahua Ni:** Writing – review & editing, Writing – original draft. **Mikhail L. Zheludkevich:** Writing – review & editing, Supervision. **Xiaonong Zhang:** Writing – review & editing, Project administration, Funding acquisition.

## Declaration of competing interest

The authors declare the following personal relationships which may be considered as potential competing interests: Xiaonong Zhang is currently employed by Suzhou Origin Medical Technology Co. Ltd..
