# Supplementary material for: High-performance Mg–Zn alloy achieved by the ultrafine grain and nanoparticle design
Source: Bioact Mater. 2024 Jul 27;41:371–84. doi: 10.1016/j.bioactmat.2024.07.020 (PMC11327953; doi:10.1016/j.bioactmat.2024.07.020)
Supplement: Multimedia component 1 [file mmc1.docx]

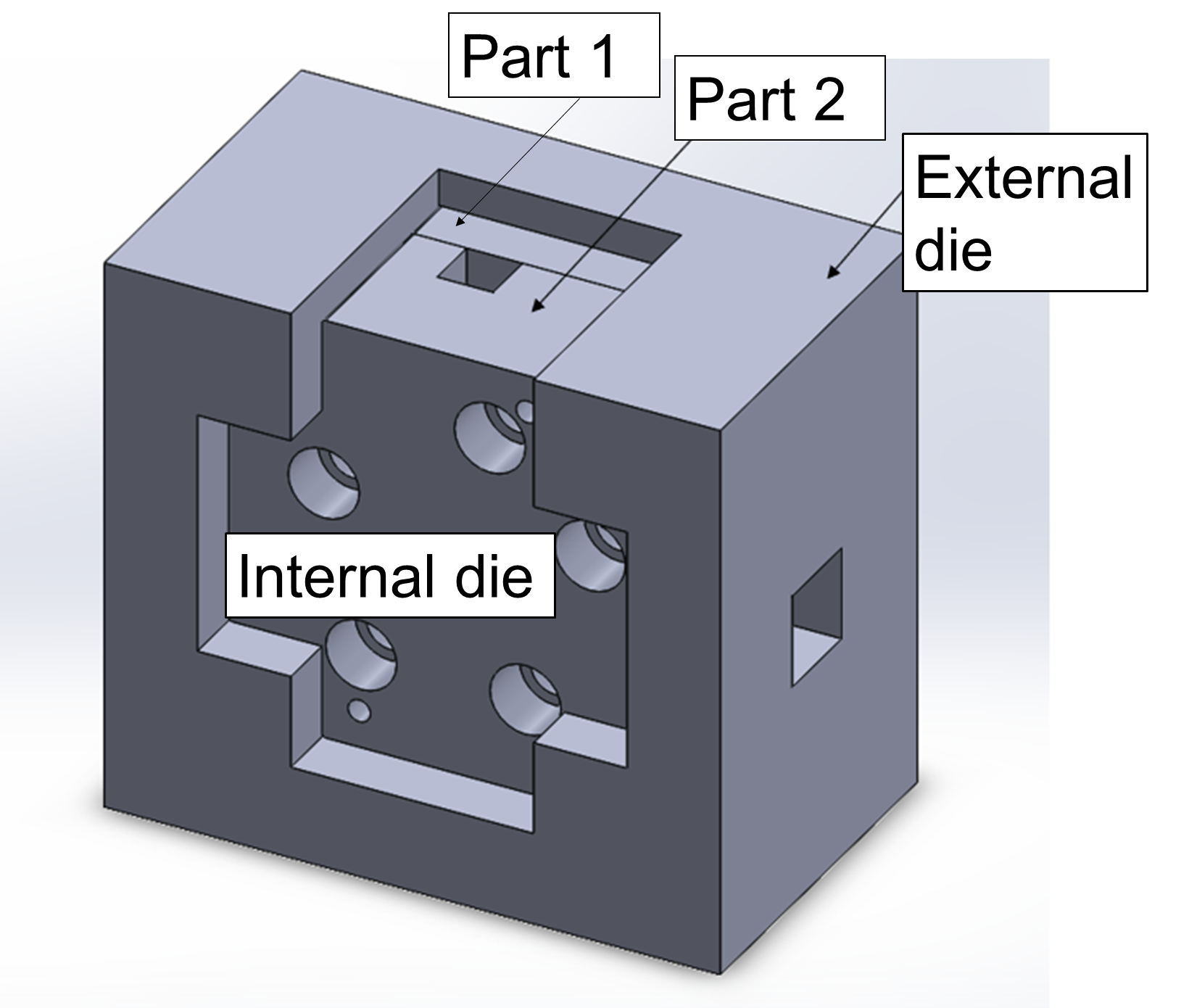


Fig. S1 The design of the ECAP die-set.

Table S1. Fitting results from the 1h EIS spectra in Hank’s solution

|  | R_s_ | CPE_1_ | | R_1_ | CPE_2_ | | R_2_ | Chi-square | R1+R2 |
| --- | --- | --- | --- | --- | --- | --- | --- | --- | --- |
|  | (Ω cm^2^) | Q_1_ | n_1_ | (Ω cm^2^) | Q_2_ | n_2_ | (Ω cm^2^) |  | (Ω cm^2^) |
|  |  | (µΩ^-1^cm^-2^s^n^) |  |  | (µΩ ^-1^cm^-2^s^n^) |  |  |  |  |
| P0 | 123.2 | 3.78E-05 | 0.8959 | 1737 | 1.21E-03 | 1 | 520.8 | 1.87E-03 | 2257.8 |
| P1 | 106.4 | 3.12E-05 | 0.8961 | 1433 | 1.23E-03 | 1 | 373.4 | 9.91E-04 | 1806.4 |
| P5 | 109.4 | 2.32E-05 | 0.8913 | 1697 | 1.42E-03 | 1 | 532.2 | 1.35E-03 | 2229.2 |
| P10 | 108 | 4.52E-05 | 0.8916 | 1448 | 2.06E-03 | 1 | 391.5 | 1.33E-03 | 1839.5 |

Table S2. Fitting results from the 24h EIS spectra in Hank’s solution

|  | R_s_ | CPE_1_ | | R_1_ | CPE_2_ | | R_2_ | Chi-square | R1+R2 |
| --- | --- | --- | --- | --- | --- | --- | --- | --- | --- |
|  | (Ω cm^2^) | Q_1_ | n_1_ | (Ω cm^2^) | Q_2_ | n_2_ | (Ω cm^2^) |  | (Ω cm^2^) |
|  |  | (µΩ^-1^cm^-2^s^n^) |  |  | (µΩ^-1^cm^-2^s^n^) |  |  |  |  |
| P0 | 142.3 | 2.18E-05 | 0.9279 | 1186 | 2.03E-03 | 0.8377 | 576.9 | 2.07E-04 | 1762.9 |
| P1 | 99.61 | 2.99E-05 | 0.8883 | 2148 | 1.42E-03 | 0.9435 | 604 | 6.59E-04 | 2752 |
| P5 | 131.7 | 3.94E-05 | 0.8822 | 1627 | 1.79E-03 | 1 | 404.4 | 4.85E-04 | 2031.4 |
| P10 | 130.3 | 3.13E-05 | 0.8973 | 2150 | 1.36E-03 | 0.8756 | 757.6 | 3.80E-04 | 2907.6 |
